# Evaluation of the radiotherapy management of ocular surface squamous neoplasia in a high HIV prevalence setting- a retrospective review

**DOI:** 10.1186/s13027-016-0064-y

**Published:** 2016-04-20

**Authors:** Ntokozo Ndlovu, Sandra Ndarukwa, Webster Kadzatsa, Simbarashe Rusakaniko

**Affiliations:** College of Health Sciences, University of Zimbabwe, Mazowe Street, Harare, Zimbabwe; Radiotherapy Centre, Parirenyatwa Hospital, Mazowe Street, Harare, Zimbabwe

**Keywords:** Ocular surface squamous neoplasia, Radiotherapy, HIV

## Abstract

**Background:**

This article evaluates a single institute’s radiotherapy management of OSSN, a previously regarded as rare malignancy, for possible future development of strategies to clearly define the role of adjuvant radiotherapy in improving treatment outcomes.

**Materials and methods:**

A retrospective review of 153 patients treated from January 2003 to December 2009.

**Results:**

There was no difference in OSSN prevalence by gender (male to female ratio 1.07). Of 80/153 patients tested 79 (98.8 %) were HIV positive. Most patients (62.9 %) had prior orbital exenteration. Moderately and poorly differentiated grade (82.3 %) was associated with significantly higher stage and incidence of positive regional lymph nodes. External beam therapy dose mostly used was 60Gy in 30 fractions at 200 cGy per fraction in 5 fractions per week (34.8 %). ^90^Strontium therapy was given to 13.5 % (60Gy in 6 fractions at 10Gy per fraction weekly). Favourable response (complete and partial) was seen in about 80 % of patients associated with higher total doses. Regional lymph node positivity was associated with poorer outcome.

**Conclusion:**

Adjuvant radiotherapy could have an important role in the management of patients presenting with locally advanced OSSN who are mostly HIV positive in developing countries. Prospective studies to evaluate the role of radiotherapy with or without chemotherapy in the management of OSSN in these settings are warranted.

## Background

Ocular surface squamous neoplasia (OSSN) has previously been considered to be a rare tumour mainly found in elderly males [1, 2]. The incidence has been increasing, especially in tropical and subtropical Africa [1]. This trend has mostly been attributed to the high prevalence of human immunodeficiency virus (HIV) and human papilloma virus (HPV) infections. High solar radiation has also been implicated in the pathogenesis of this disease in this region [3–6].

It has now become known as aggressive, affecting young people equally for both genders [4]. In one study, 97 % of patients were found to be HIV positive, a suggestion that ocular surface squamous neoplasia was an AIDS-defining neoplasm with high morbidity and mortality [7].

The aggressiveness of this disease has been associated with late stage disease requiring more aggressive surgery in the form of enucleation and exenteration [8]. The local recurrence rates for ocular surface squamous neoplasia have been shown to be as high as 43 %, thus confirming the need for consideration of adjuvant radiotherapy in this disease, even if the margins of excision are negative [1, 9].

There have not been any randomised controlled clinical trials reported in the literature on the management of this disease, whether by radiotherapy or other means [1]. Most of the reports in the literature pertaining to the management of this disease are case reports and most of those on radiotherapy treatment of ocular surface squamous neoplasia describe strontium 90 therapy [10, 11].

In this article a review of the radiotherapy management of ocular surface squamous neoplasia including external beam therapy over a six-year period during high HIV prevalence is presented. This is one of the largest series of patients with this disease to be reported with 153 patients. The disease characteristics in relation to histological features and stage of the disease in patients presenting with ocular surface squamous neoplasia are presented. The radiotherapy management of patients with the disease is evaluated and information on the outcome of management is provided. Recommendations for the management of ocular surface squamous neoplasia are also made based on results obtained.

The aim of the research reported in this article was to assist in developing a better understanding of ocular surface squamous neoplasia presenting with HIV disease in order to develop prospective research strategies that will contribute to clarifying the role of radiotherapy management of this disease and improving of outcomes of treatment in the future.

## Methods

This paper reports on a retrospective review of patients who consecutively presented at the Parirenyatwa Radiotherapy Centre, Harare, Zimbabwe. Radiotherapy treatment records of patients presenting in the period between 1 January 2003 and 31 December 2009 with a diagnosis of OSSN were reviewed.

All patients presenting with histologically confirmed OSSN during that period and were above the age of 18 years were eligible for participation in the study. Prior to presentation, surgical intervention in the form of incisional biopsy, simple local excision, enucleation or exenteration (partial and total) had been performed on the patients as surgical management.

All patients without a histologically proven diagnosis of SCC conjunctiva, patients under the age of 18 years and patients referred without adequate required documentation were excluded. The information was obtained from patient hospital records.

Outcome of treatment was recorded according to whether there was complete response (no disease detected clinically), partial response (good/favourable response), stable disease (no change to size/symptoms of disease) or disease progression (a notable increase in size of disease or new lesions elsewhere) to treatment measured clinically at follow up.

Follow up data was collected from the first documented visit after completion of treatment. All information from the patient records were extracted on to a designed extracted form. All data collected was quality controlled for completeness of information through crosschecking by at least 2 individuals. The data was entered into EPI Info 3.5.3. All data entered was quality controlled for data entry errors using SPSS version 16 and Epi Info 3.5.3. Data was then imported and analysed in STATA version 10 and SPSS version 16.

### Statistical analysis

Descriptive statistics were used to describe demographic and disease characteristics. Trends were used to describe the number of patients referred to the radiotherapy centre over time and socio-demographic characteristics over time. Chi-square tests were used for trend significance and for association between categorical variables. Statistical significance was defined at a p value of <0.05.

#### Ethical considerations

This research was carried out in accordance with the principles of the Declaration of Helsinki. The research protocol was submitted for consideration, comment and guidance to the Joint Parirenyatwa Hospital and College of Health Sciences Research Ethics Committee (JREC) and was approved by that ethics body prior to conducting the research.

## Results

In all, 153 patients’ records with this diagnosis were retrieved and formed the study population. The patients’ demographic characteristics, surgical/radiotherapy management, response to treatment and follow up will now be discussed.

Out of the total of 153 patients, 79 (51.6 %) were male and 74 (48.4 %) were female (male to female ratio 1.07). The median age of the patients at presentation was 40 years with an interquartile range (IQR) of 36-48 years. There was no statistically significant difference between the number of male and females (*p* = 0.80) and this remained so over the years of the study. The numbers of patients seen showed an upward trend with relation to time over the years as shown by the chi square test for trend analysis (*p* = 0.03). Married patients constituted 78 (51.6 %) and 43 (28.1 %) of all the patients were widowed.

Table 1 summarizes the demographic characteristics of the patients.

**Table 1:** Demographic characteristics of OSSN patients

Characteristic	*N* = 153	Percentage
Gender		
Male	79	51.6 %
Female	74	48.4 %
Marital Status		
Single	13	8.5 %
Married	78	51.6 %
Separated	8	5.2 %
Divorced	11	7.2 %
Widowed	43	28.1 %
Place of Residence		
Rural	69	45,1 %
Peri-urban	11	7.2 %
Urban	73	47.7 %
Employment status		
Unemployed	101	66.7 %
Informally employed	24	15.7 %
Formally employed	27	17.65

There was no difference in the proportions of rural resident (47.7 %) and urban resident urban (45.1 %) patients with OSSN. No gender differences were demonstrated between these 2 population groups.

### HIV and antiretroviral (ART) status

The HIV status was established in 81/153 (53.0 %) of these patients. The HIV positivity rate was 98.8 % (80/81). Of the patients who tested positive, the CD4 count was established in only 37 patients. The median CD4 count was 122 cells per micro-litre with an interquartile range (IQR) of 84-182 cells pre micro-litre.

The HIV status was unknown in 72/153 (47.1 %). Of the patients with an unknown status 21/72 (29.10 %) had either stigmata of HIV infection or reported having an HIV-infected husband or young children alive or who had died from an HIV-related illness.

Of the patients who were HIV positive only 34/80 (42.5 %) were on antiretroviral therapy (ART) at presentation. Whilst none of the known HIV positive patients were on ART at the start of the study in 2003, 13/25 (52 %) of known HIV positive patients were on ART by 2009. The median duration on ART prior to presentation was 12 weeks with an interquartile range (IQR) of 3-24 weeks.

### Surgery and disease characteristics

Table 2 summarises the surgical interventions and pathology findings in the patients.

**Table 2:** Surgery and pathologic findings

Characteristic	*N* = 153	Percentage
Type of surgery		
Biopsy	10	6.6
Simple excision	32	20.9
Enucleation	15	9.8
Exenteration	90	58.8
Partial exenteration	6	3.9
Histology		
Carcinoma in situ	5	3.3
Well differentiated	22	14.4
Moderately differentiated	92	60.1
Poorly differentiation	34	22.2
Stage		
I	31	20.3
II	30	19.6
III	51	33.3
IV	41	26.8
Regional lymph nodes		
Positive	41	26.8
Negative	112	73.2
Simple excision margins		
Negative	0	0
Close	6	18.8
Positive	17	53.1
Unknown	9	28.1
Enucleation/Exenteration		
Margins		
Negative	2	1.8
Close	33	29.7
Positive	46	41.5
Unknown	30	27.0

The commonest form of surgery performed was exenteration with 90 patients (58.8 %) having had total exenteration and 6 (3.6 %) patients having had partial exenteration performed.

The common histological grades in 82.3 % of the study patients were moderately and poorly differentiated squamous cell carcinoma. Overall, 2 (1.3 %) patients were found to have squamous cell carcinoma in the background of pterygium and 2 (1.3 %) patients in the study had a spindle cell variant of squamous cell carcinoma.

Patients with poorly differentiated histological grade were more likely to present with advanced stage disease whereas those with other histological grades presented with early stage disease (*p* = <0.001). Out of the 87 patients with stage 3 disease, 82 (94.3 %) had moderately or poorly differentiated tumours.

Most of the 32 patients (17 or 53.1 %) who had simple surgical excisions had positive margins whilst 39 (25.0 %) had unknown margins. There appeared to be no relationship between the type of surgery and status of margins of excision.

Of the 41 patients with positive regional lymph nodes 38 (90.2 %) had stage III and IV disease. Poorer histological grades were associated with the presence of positive lymph nodes (see Table 3). Clinical evaluation for evidence of gross tumour in the orbit and regional lymph nodes exhibited clinically evident disease in 82/153 (53.6 %) patients.

**Table 3:** Histological grade versus regional lymph node (RLN) status

Histological grade	Regional lymph nodes present	Regional lymph nodes absent	Total
Carcinoma in situ	0 (0 %)	5 (4.5 %)	5 (3.5 %)
Well differentiated	2 (4.9 %)	20 (17.9 %)	22 (14.4 %)
Moderately differentiated	25 (61 %)	67 (59.8 %)	92 (60.1 %)
Poorly differentiated	14 (34.1 %)	20 (17.9 %)	34 (22.2 %)
Total (% of total)	41 (25 %)	112 (75 %)	153 (100 %)

*p* value 0.03

### Patient treatment

Following initial evaluation 104/153 (67.97 %) patients were offered treatment. Of those not offered treatment, 25/49 (16.3 %) defaulted review following the initial consultation, 18/49 (11.8 %) were referred for further surgery and were subsequently lost to follow-up. Six patients (3.9 %) were lost to follow-up due to time delay before starting treatment.

Of the 104 patients who received treatment, 89 (84.6 %) were prescribed external beam therapy whilst 14 (13.5 %) were prescribed strontium 90 therapy. Only one patient was offered best supportive care.

Of those who received treatment 59 (56.7 %), patients were considered for treatment with radical/curative intent and 45 (43.3 %) patients were considered for palliative treatment. Of patients prescribed radiotherapy treatment 8 (7.8 %) failed to commence the treatment.

All 14 patients who were treated with strontium eye applicator were prescribed 60 Gray (Gy) in six fractions in six weeks (10 Gy per fraction weekly).

Table 4 summarises the doses prescribed for external beam radiotherapy. External beam radiotherapy was comprised of conventional 2D treatment.

**Table 4:** Prescribed doses for external beam

Prescribed dose ^a^	Frequency	Percentage
Single fraction 600 cGy	1	1.1
30 Gy in 10 fractions at 300 cGy/fraction	22	24.7
37.50 Gy in 15 fractions at 250 cGy/fraction	3	3.4
45 Gy in 20 fractions at 225 cGy/fraction	2	2.2
50 Gy in 25 fractions at 200 cGy/fraction	6	6.7
50 Gy in 20 fractions at 250 cGy/fraction	16	18.0
60 Gy in 30 fractions at 200 cGy/fraction	31	34.8
Other	8	9.0
Total	89	100

Key – ^a^except for single fraction treatment. Treatment was given five days per week

The commonest prescribed dose was 60 Gy in 30 fractions in 6 weeks (200 cGy per fraction). This was prescribed for 31/89 (34.8 %) of the patients. Only one patient was prescribed a single fraction of 600 cGy. The other common prescriptions included 40 Gy in 15 fractions in 3 weeks (266 cGy per fraction), 20 Gy in 5 fractions in 1 week (400 cGy per fraction) and 66 Gy in 33 fractions delivered over 6½ weeks (200 cGy per fraction).

Anterior and lateral wedged pair orbital fields were used to treat 77/89 (86.0 %) patients receiving external beam radiotherapy. Ten patients (11.2 %) were treated with a single anterior field either as a direct anterior or anterior oblique field. The remaining 2 patients were treated with non-standard fields with palliative intent due to extensive disease.

Of the 103 patients who were prescribed and commenced radiotherapy 14 (13.6 %) did not complete treatment. Of these 13 absconded, whilst only 1 had treatment discontinued due to deterioration in performance status. Of the 81 patients who received radiotherapy 78 (96.29 %) completed within the prescribed time.

A total of 78 patients presented for follow up post-treatment. The median follow-up was 6 weeks (IQR 6-12 weeks). Of these patients 41 (52.6 %) had presented with clinical disease and 37 (47.4 %) patients had not.

When the patients with clinical disease at the start of treatment were assessed for response to treatment at follow-up, 9/41 (22.0 %) had a complete response and 28/41 (68.3 %) had partial response. Disease progression was seen in 4 (9.7 %) patients. The follow-up time ranged from 2-52 weeks.

The majority of patients presenting for follow up (82.9 %) had been treated with palliative intent. Four (11.8 %) of these patients achieved a complete response. Doses prescribed for these patients were 30 Gy in 10 fractions at 300 cGy per fraction 5 days per week (n = 2), 50 Gy in 20 fractions at 250 cGy per fraction 5 days per week (n = 1) and the other 50 Gy in 25 fractions at 200 cGy per fraction 5 days per week. Five (71.4 %) patients treated with a curative intent achieved a complete response and all 5 had been prescribed 60 Gy in 30 fractions at 200 cGy per fraction.

The presence of regional lymph nodes was associated with a poorer response to external beam radiotherapy (see Table 5). Of the 23 patients who had regional lymph node involvement, only 2 (8.7 %) had a complete response to treatment. Seventeen (73.9 %) had a partial response whilst 4 (17.4 %) had disease progression. There was a statistically significant association between a positive regional lymph node status and poor response rate (*p* = 0.002).

**Table 5:** Regional lymph node (RLN) status versus clinical response to treatment

RLN	Response rate			
	Complete	Partial	Progression	Total
Present	2 (8.7 %)	17 (73.9 %)	4 (17.4 %)	23 (100 %)
Absent	7 (38.9)	11 (61.1 %)	0 (0.0 %)	18 (100 %)
Total	9	28	4	41

A favourable response (complete and partial) for the patients with clinical initially evident disease was associated with higher total dose of radiotherapy delivered. Doses above 37.5 Gy in 15 fractions delivered at 250 cGy in 3 weeks achieved either a complete response or partial response (see Table 6).

**Table 6:** Response rate of clinically detected disease by total dose delivered

	Response rate			
Total dose delivered ^a^	Complete	Partial	Progression	Total
Single fraction	0	0	1	1
30 Gy in 10 #	2	15	1	18
20 Gy in 5 #	0	1	2	3
37.5 Gy in 15 #	0	2	0	2
45 Gy in 20 #	0	1	0	1
40 Gy in 15 #	0	1	0	1
40 Gy in 20 #	0	1	0	1
50 Gy in 20 #	1	1	0	2
50 Gy in 25 #	1	1	0	2
54 Gy in 27#	0	1	0	1
60 Gy in 6 #^b^	2	0	0	2
60 Gy in 30 #	3	4	0	7
Total	9	28	4	41

Key - ^a^ # = fractions given at 5 fractions per week; ^b^ patients who received strontium therapy

Of the patients presenting without clinical disease 35(91.6 %) were free of recurrence and 2 (5.4 %) had disease recurrence during follow-up. The maximum follow-up was 60 weeks and the minimum follow-up was 2 weeks.

Overall, these results pertained to this population of relatively young, mostly HIV positive patients who had advanced OSSN treated postoperatively mainly with external beam radiotherapy and with Strontium therapy. Results of treatment were largely favourable with favourable response associated with higher radiotherapy doses. Regional node positivity was associated with a poorer response to treatment. Post treatment follow up was not optimal.

## Discussion

OSSN is the most common malignancy of the conjunctiva [4]. Its association with HIV infection as well as other causes of immunosuppressive disorders such as has been demonstrated in transplant patients and systemic lupus erythematosus (SLE) is documented [8, 10, 12]. Current clinical practice for the treatment of OSSN is based on a weak evidence base. This evidence has been largely attained from case reports and case series [1].

The results were in keeping with a lower age at presentation and lack of male preponderance that has been demonstrated in other settings of high HIV infection prevalence [5, 13]. In this research the HIV positivity rate was 98.8 % in those patients who were tested. This is in keeping with previously reported rates in the region. Nearly half of the patients in this study were not tested for HIV due to non-mandatory testing being the norm during the study period. It has however been clearly demonstrated that an HIV prevalence above 90 % is common in this cancer [4, 7]. The impact of the use of ART on the natural history of the disease in these patients remains to be seen. This research was carried out during a period of low ART uptake.

It has been previously shown, as in this research, that larger tumours such as those seen in patients with HIV infection tend to be invasive, need more extensive surgery, and frequently recur after surgery [4, 8, 14, 15]. This raises the question of what adequate initial surgery should be performed and what criteria to use to initiate adjuvant radiotherapy or other therapies following surgical intervention [9, 14].

Most patients used as a sample in this research had advanced stage and moderately or poorly differentiated tumours. These characteristics are likely to account for the high rate of enucleation and exenteration (72.5 % combined). An increase the likelihood of local recurrence following surgery has been previously demonstrated [14]. If it is the case this further builds on to the need for postoperative radiotherapy.

Management of regional lymph nodes in OSSN is yet to be clearly defined. In this research, the association between poorer differentiated and involved lymph nodes may suggest the need to explore what would be adequate nodal management in OSSN by stage and grade of tumour. Sentinel lymph node biopsy may be one such approach [16].

Whilst this research reports on postoperative radiotherapy treatment of OSSN other modalities of treatment have been considered. At the centre chemotherapy was not used post-operatively. Documented interventions include immunotherapy [8, 17], photodynamic therapy [18], and topical chemotherapy [9, 19].

Reports on the radiotherapy management of OSSN in the literature are mostly on strontium 90 therapy for superficial lesions [10, 11]. A number of dose and fractionation schedules have been previously reported for strontium 90 therapy, with some authors giving doses as high as 60 Gy in a single fraction and 140 Gy in 7 fractions [10]. In this research, 60 Gy at 10 Gy per fraction per week in 6 weeks was used for all patients receiving strontium 90 treatment.

Few case series have been published on the use of other radiotherapy modalities including electron external beam treatment [20] and plaque brachytherapy [21]. Electron beam radiotherapy has been used in one reported case to preserve eyesight in recurrent disease [22]. Similarly in another case report proton beam therapy was used in 2 cases of extensive disease preoperatively [23].

The more frequent use of external beam therapy compared with strontium 90 reported in this research (84.6 % versus 13.5 %) can be attributed to the dictates of the disease characteristics in our patients. In this research, photon external beam treatment was given using general guidelines for the use of orbital treatment fields.

This retrospective study that was carried out in a low resource setting had limitations, one being poor follow up of patients after treatment. This limited the acquisition of information on long-term outcomes and treatment complications.

Some of the findings of this study are in keeping with what has been documented from similar high HIV prevalence settings. The use of external beam radiotherapy in similar patients has however not been commonly reported.

## Conclusion

Radiotherapy treatment in OSSN – and in a high HIV prevalence environment particularly, has a role to play due to the tumour characteristics in such patients. This is clearly demonstrated in the research reported in this article by the high overall response rate to this treatment. With OSSN increasingly affecting young adults and causing substantial morbidity and mortality, it becomes important to further study what the optimum interventions are.

There currently is no evidence based practice guideline on the management of this disease in both HIV positive and negative patients. Most clinical management is based on case reports. This is one of the largest series of patients with OSSN reported in the literature and it has shown the positive impact of radiotherapy in advance disease.

There is a significant association between HIV infection and development of OSSN. Radiotherapy could play an important role in the management of patients presenting with locally advanced disease. Prospective studies could establish the role of radiotherapy with or without chemotherapy and regional lymph node surgery in this patient population.
